# The impact of global budget on expenditure, service volume, and quality of care among patients with pneumonia in a secondary hospital in China: a retrospective study

**DOI:** 10.1186/s12889-020-08619-3

**Published:** 2020-04-19

**Authors:** Xiaodong Guan, Chi Zhang, Huajie Hu, Luwen Shi

**Affiliations:** 1 Department of Pharmacy Administration and Clinical Pharmacy, School of Pharmaceutical Sciences, Peking University, Beijing, 100191 China; 2 International Research Center for Medicinal Administration, Peking University, Beijing, China

**Keywords:** Global budget, Fee for service, Daily expenditure, Service volume, Quality of care

## Abstract

**Background:**

The Chinese government has begun to dampen the growth of health expenditure by implementing Global Budgets (GB). Concerns were raised about whether reductions in expenditure would lead to a deterioration of quality of care. This paper aims to evaluate the impact of GB on health expenditure, service volume and quality of care among Chinese pneumonia patients.

**Methods:**

A secondary hospital that replaced Fee-For-Service (FFS) with GB in China in 2016 was sampled. We used daily expenditure to assess health expenditure; monthly admission, length of stay (LOS), number of drugs per record and record containing antibiotics to evaluate service volume; record with multiple antibiotics and readmission to assess quality of care. Descriptive analyses were adopted to evaluate changes after the reform, logistic regression and multivariable linear regressions were used to analyze changes associated with the reform.

**Results:**

In 2015 and 2016, 3400 admissions from 3173 inpatients and 2342 admissions from 2246 inpatients were admitted, respectively. According to regression analyses, daily expenditure, LOS, readmission, and records with multiple antibiotic usages significantly declined after the reform. However, no significant relation was observed between GB and the number of drugs per record or record containing antibiotics.

**Conclusions:**

When compared with FFS, GB can curtail health expenditure and improve quality of care. As far as service volume was concerned, LOS and monthly admission declined, while number of drugs per record and record containing antibiotics were not affected.

## Background

There is a global trend towards increases in healthcare expenditure. As the World Health Organization (WHO) notes the average annual rate of global health expenditure increased 4.0% from 2000 to 2015, surpassing the global economy by 1.2% [1]. This situation has put pressure on healthcare reimbursement –– to the alarm of many governments –– leading to reimbursement reform from traditional Fee-For-Service (FFS) to Global Budget (GB). GB prospectively sets caps on spending for hospitals, physicians and pharmaceutical firms [2]. Traditional FFS is facing many challenges as it contains motivations to increase the volume and cost of services, duplication, and inefficiency in the delivery of medical services [3]. Conversely, GB showed far greater control over health expenditures with its inherent financial risk for health care providers [4–6]. Once the total expenditure exceeds the pre-set expenditure, the government shall not earmark funds to the hospital and the hospital must take responsibility for its own profits and losses.

Healthcare expenditure in China has been rising since economic reform in 1978, and the annual growth rate was 12.5%, exceeding the 9.9% economic growth rate of the same period from 1993 to 2012 [7]. To control healthcare expenditure from fast growing, the Chinese government initiated GB reforms in place of FFS system in 2009 [8].

GB often defrays the total expenditure of the coming year according to outpatient amount and number of patients discharged in previous years, considering the price index [9, 10]. This system pushed many hospitals to curb spending. There are two alternative methods with which hospitals manage to curb health expenditure in empirical studies. One is a retrospective price-setting mechanism based on past gross expenditure to set an expenditure cap [11, 12]. With the restriction of daily expenditure or total cost per record, the total expenditure can be controlled. The other system is to control service volumes [12–17] such as length of stay (LOS), hospital visits, drug utilization, and other services. For instance, the Chinese government once implemented a policy restricting the proportion of drug costs accounting for total medical expenditure to 30%, leading to a decrease in the utilization of drugs [18].

However, studies evaluating the impacts of GB on expenditure and service volume observed mixed results. While most researchers claimed that GB curtailed the growth of health expenditure, Lee and Jones [19] found that daily expenditure increased as GB was implemented in Taiwan, 2004. Lin et al. [20] observed significant increases in service volume in Taiwan, 2016, while Markovich et al. [15] claimed that service volume decreased in California, 2012. Factors such as changes in service volume might affect quality of care, [13, 16, 21] and the overall impact of GB on quality of care is not yet clear.

In this study, a secondary hospital located in Yunnan Province, southwest China was selected as the sample hospital in our before-and-after design. In 2015, the hospital had 660 beds, 306,949 visits and 16,359 admissions. In January 2016, this hospital implemented a payment reform where GB substituted for FFS among inpatients. In 2002, the Chinese government decided to re-establish its rural health insurance scheme, the New Rural Medical Cooperative Scheme (NCMS), with financial support from both central and local governments [12]. The financial support from governments accounted for around 80% of the total fund, and all the Chinese rural residents were eligible to enroll in the scheme. Over the past decade, urban employee basic medical insurance (UEBMI), a social health insurance scheme jointly funded by employers and employees. Meanwhile, another health insurance scheme mainly funded by the government and residents called urban resident basic medical insurance (URBMI) also developed. URBMI have also been strengthened, covering more employees from public and private sectors than ever. For NCMS system, UEBMI and URBMI enrollee, the local government negotiates budgets with hospitals at the end of each year and signs contracts to determine the total budget of the coming year, taking revenues and expenditure, total budget limits, and sharing risk into consideration. We hypothesized that the implementation of GB could save health expenditure, reduce service volume and improve quality of care.

## Methods

### Data source

The sample hospital lies in a relatively under-developed province in China as a secondary public hospital, and around 160 thousand inpatients are admitted to it annually. Inpatient records from internal medicine and pediatric departments whose diagnosis contained pneumonia were extracted from the Hospital Information System from January 1st, 2015 to December 31st, 2016. Collected information comprised patients’ ID, admission number, department, diagnosis, therapeutic effects, rescue effects, name of doctor, total expenditure, specification, unit price and the quantity of drugs prescribed. The policy came into effect on January 1st^,^ 2015, so we divided patients into before-intervention group and after-intervention group according to the day they discharged.

### Measurement indicators

This research aimed at evaluating the impact of payment reform on expenditure, service volume and quality of care. We chose daily expenditure per person for expenditure assessment; monthly admission, LOS, number of drugs per record and records containing antibiotics to evaluate service volume; records with multiple antibiotics together with readmission within 30 days to assess quality of care.

### Statistical analysis

Univariate analysis was adopted to analyze changes of covariates and dependent variables before and after the reform. Furthermore, age group, sex and department were included as covariates in the multivariable linear regression and logistic regression (Supplementary Data). Multivariable linear regression was employed to analyze the impact of reform laid on daily expenditure, LOS, and number of drugs per record, while logistic regression was adopted to evaluate the impact that the reform had on records containing antibiotics, records with multiple antibiotics and readmission.

The equation of multivariable linear regression is presented below:

$$ {\mathrm{Y}}_{\mathrm{t}}={\upbeta}_0+{\upbeta}_1\ast {\mathrm{x}}_1+{\upbeta}_2\ast {\mathrm{x}}_2+{\upbeta}_3\ast {\mathrm{x}}_3+{\upbeta}_4\ast {\mathrm{x}}_4+{\upvarepsilon}_{\mathrm{kt}} $$

X_1 ~ 4_ were variables indicating whether the reform took place, age group, sex and department of the patients. β_0_ was the constant, and *ε*_*kt*_ stood for error.

Equation of logistic regression is presented below:

$$ \Pr \left(\mathrm{Y}=1|\mathrm{x}\right)=\mathrm{p}\left(\mathrm{x}\right)=\frac{\exp \left({\upbeta}_{5+}{\upbeta}_6{\mathrm{x}}_6+{\upbeta}_7{\mathrm{x}}_7+{\upbeta}_8{\mathrm{x}}_8+{\upbeta}_9{\mathrm{x}}_9\right)}{1+\exp \left({\upbeta}_{5+}{\upbeta}_6{\mathrm{x}}_6+{\upbeta}_7{\mathrm{x}}_7+{\upbeta}_8{\mathrm{x}}_8+{\upbeta}_9{\mathrm{x}}_9\right)} $$

Pr indicated probability, β_5_ was the constant, x_6 ~ 9_ were variables indicating whether the reform took place, age group, sex and department of the patients, β_6 ~ 9_ were coefficients corresponding to x_6 ~ 9_.

In this study, *p* value less than 0.05 was regarded as a significant indication.

## Results

### Patient demographics

3400 admissions were included in the 2015 unit and 2342 in the 2016 unit, which can be attributed to 3173 and 2246 patients respectively. In the pre-reform and post-reform groups, 36.7 and 28.2% were under 5 years of age, 15.5 and 14.0% were aged from 5 to 18, 23.2 and 28.5% were aged from 18 to 65, 24.6 and 29.2% were over 65 years old. When classified by department, patients from internal medicine accounted for 48.5% of pre-reform inpatients and that of post-reform inpatients was 58.7% (*p* < 0.001). Among pre-reform and post-reform patients, 56.4 and 55.6% were male, respectively (Table 1).

**Table 1:** Patient demographic characteristics

	Study sample(***N*** = 5419)	
Characteristics	Pre-reform (***N*** = 3173)	Post-reform (***N*** = 2246)
**Age group**		
[0,5)	1164 (36.7%)	634 (28.2%)
[5,18)	492 (15.5%)^***^	315 (14.0%)^***^
[18,65)	736 (23.2%)^***^	641 (28.5%)^***^
[65, 95]	781 (24.6%)^***^	656 (29.2%)^***^
**Department**		
Pediatric	1635 (51.5%)^***^	928 (41.3%)^***^
Internal medicine	1538 (48.5%)^***^	1318 (58.7%)^***^
**Sex**		
Male	1790 (56.4%)	1249 (55.6%)
Female	1383 (43.6%)	997 (44.4%)

*p < 0.05, **p < 0.01, ***p < 0.001

### Expenditure changes before and after the GB reform

Daily expenditure of pre-reform inpatients and post-reform inpatients did not differ significantly from Wilcoxon rank sum test (Table 2). However, after controlling for variations in sex, age, and department, a significant negative correlation between daily hospitalization expenditure and reform was observed (β_1_ = − 0.017, *p* < 0.001, Table 3).

**Table 2:** Descriptive analyses of expenditure, service volume and quality of care

	Total sample(***N*** = 5742)	
Variable	Pre-reform(***N*** = 3400)	Post-reform (***N*** = 2342)
**Log transformed daily expenditure**		
Median value^w^	2.68	2.68
**Monthly admission**^w^		
	284	208.5^***^
**LOS**^w^		
Median value	7	8^***^
**Number of drugs per record**^w^		
Median value	15	15
**Record containing antibiotics**^c^		
Yes	3101 (91.2%)	2114 (90.3%)
No	299 (8.8%)	228 (9.7%)
**Readmission**^c^		
Yes	142 (4.2%)	66 (2.8%)
No	3258 (95.8%)^**^	2276 (97.2%)^**^
**Records with multiple antibiotics**^c^		
Yes	1105 (32.5%)	653 (27.9%)
No	2295 (67.5%)^***^	1689 (72.1%)^***^

*p < 0.05, **p < 0.01, ***p < 0.001

^w^Wilcoxon rank-sum test

^c^Chi-square test

**Table 3:** Results of multiple linear regression analyzing log transformed daily expenditure, LOS and number of drugs per record

	Log transformed daily expenditure	95% CI interval	LOS	95% CI interval	Number of drugs per record	95% CI interval
Post-reform	−0.017^***^	− 0.023 to − 0.010	−0.475^***^	− 0.693 to − 0.256	−0.176	− 0.498 to 0.146
Age group						
[5,18)	0.016^**^	0.006 to 0.026	0.503^**^	0.172 to 0.835	0.780^**^	0.292 to 1.267
[18,65)	0.003	−0.037 to 0.043	4.120^***^	2.823 to 5.418	3.472^***^	1.563 to 5.381
[65, 95]	0.038	−0.002 to 0.078	6.378^***^	5.082 to 7.675	9.289^***^	7.381 to 11.197
Female	−0.002	−0.008 to 0.005	− 0.300^**^	−0.515 to − 0.084	−0.104	− 0.422 to 0.213
Internal medicine	0.127^***^	0.088 to 0.165	1.601^*^	0.335 to 2.868	−1.883^*^	−3.746 to −0.019

Reference groups were pre-reform, [0, 5), male and pediatric, respectively

*p < 0.05, **p < 0.01, ***p < 0.001.

### Service volume changes before and after the GB reform

The median monthly admission of pre- and post-reform inpatients differed significantly (*p* < 0.001, Table 2).

The median LOS of pre- and post-reform inpatients differed significantly (*p* < 0.001, Table 2). However, the results of multivariable linear regression showed that the reform was in significant negative correlation with LOS when covariates were adopted in the analysis (β_1_ = − 0.475, *p* < 0.001, Table 3).

Numbers of drugs per record for inpatients before and after the reform did not differ significantly in accordance with Wilcoxon rank sum test (Table 2). No significant correlation between the reform and numbers of drugs per record was observed in multivariable linear regression, either (Table 3).

As for records containing antibiotics, the Chi-square test did not produce evidence of any significant change between patients admitted in 2015 and 2016 (Table 2). Regarding the covariates, the reform did not significantly correlate with records containing antibiotics according to logistic regression (Table 4).

**Table 4:** Results of logistic regression analyzing record containing antibiotics, readmission and records with multiple antibiotics

Variable	Total sample(***N*** = 5742)		
	Standard error	OR value	95% CI interval
**Record containing antibiotics**			
Post-reform	0.11	1.11	(0.92, 1.34)
Age group			
[5,18)	18.78	18.55^**^	(2.55, 134.89)
[18,65)	13.18	12.04^*^	(1.41, 102.97)
[65, 95]	32.50	29.64^**^	(3.46, 254.21)
Female	0.10	1.04	(0.86, 1.26)
Internal medicine	0.01	0.01^*^	(0.00, 0.05)
**Readmission**			
Post-reform	0.62	0.62^**^	(0.46, 0.84)
Age group			
[5,18)	1.58	1.58	(0.99, 2.52)
[18,65)	2.95	2.95	(0.38, 23.05)
[65, 95]	3.42	3.42	(0.44, 26.60)
Female	0.15	1.01	(0.77, 1.34)
Internal medicine	0.70	0.68	(0.09, 5.12)
**Records with multiple antibiotics**			
Post-reform	0.05	0.77^***^	(0.68, 0.86)
Age group			
[5,18)	0.11	1.20^*^	(1.01, 1.44)
[18,65)	0.28	0.84	(0.44, 1.61)
[65, 95]	0.35	1.06	(0.56, 2.03)
Female	0.07	1.13^*^	(1.01, 1.27)
Internal medicine	0.51	1.60	(0.85, 3.00)

Reference groups were pre-reform, [0, 5], male and pediatric, respectively

*p < 0.05, **p < 0.01, ***p < 0.001

### Quality of care changes before and after the GB reform

Compared with inpatients from 2015, readmissions of inpatients from 2016 significantly differed (*p* < 0.01, Table 2) according to chi-square test. Logistic regression was undertaken and a significantly negative correlation was observed (OR = 0.62, *p* < 0.01, Table 4).

The result of the chi-square test demonstrated that records with multiple antibiotics significantly changed after the reform (*p* < 0.001, Table 2). With the covariates used, logistic regression tested out significant negative correlations between the reform and records with multiple antibiotics (OR = 0.77, *p* < 0.001, Table 4).

## Discussion

Our results confirmed major findings that GB can curb health expenditure, as previous literature indicated [15, 21, 22]. Multivariable linear regression found a significant negative correlation between the reform and daily expenditure, and we still accepted the assumption that GB can curtail the growth of health expenditure. Under pressure to control expenditure from the government, the sample hospital pursued a strategy to set annual budgets for every department, which could be the main reason for its success. When compared to relevant research, we found that GB showed sufficient ability to curtail health expenditure in Chinese mainland hospitals, [6, 12, 17] whilst GB failed to control health expenditure and improve quality of care in Taiwan [23]. Thus we inferred that the effects of implementing GB were relevant with the method of implementation and the intrinsic structure where GB was imposed.

The results also revealed that LOS descended significantly, which could be another explanation for its success. Similarly, Markovich et al. [15] found that the savings after implementing Global Budget stemmed from declines in inpatient LOS and thirty-day readmission. According to descriptive analyses, monthly admissions significantly declined after the reform, which was consistent with previous literature that service volume would decrease under GB system [16, 24]. It might be explained that under the pressure of growing health expenditure, a portion of Chinese hospitals refused to treat patients with NCMS, UEBMI or URBMI, [24] and this issue really raised our concerns.

The number of drugs per record as well as records containing antibiotics did not change significantly after the reform. One possible reason might be that 60% of pneumonia patients were children and elders whose therapeutic regimen mainly consisted of necessary treatments even under FFS payment system. Therefore, when FFS was substituted with GB, only unnecessary treatments could be restrained to curb health expenditure. For example, expensive “hot drugs” were substituted for inexpensive drugs [23]. In this way, the number of drugs per case and records containing antibiotics were not affected while the expense was well controlled.

Improvements concerning readmission and records with multiple antibiotics were observed. It is thought that record containing multiple antibiotics are unnecessarily common in China, [25] and multiple antibiotics are associated with drug resistance [26]. Declines in record containing multiple antibiotics in this study indicates that quality of care might have been improved to some extent. We paid special attention to this result as previous research in China observed significant positive correlations between GB and readmission, [23] whilst that is not the case in other regions [21, 22]. Reductions in readmission may be attributed to the following fact that a patient without further readmission indicates a satisfactory effect of the treatment to a large extent [22].

This research evaluated the impact of GB on quality of care, which was in line with current trend of analyzing quality outcomes [11]. As Schroeder and Frist [3] noted, quality measures were necessary to ensure that evidence-based care was not denied as a cost-saving mechanism. A body of evidence now showed that rational use of drugs not only would save money but also would lead to better quality of service outcomes. In light of this, we recommend including quality of care as an index to evaluate effects brought by GB, in this way not only is quality guaranteed, but total expenditure is reduced, so negative impacts on quality of care brought by GB can be diminished.

### Limitations

This study contains several limitations. Firstly, expenditure adopted in this research was total expenditure, while reimbursement expenditure was not included, hence the effect of GB on reimbursement expenditure was not precisely represented. Secondly, data was retrieved from the same hospital, and readmission might be underestimated. Considering the city’s low population mobility and the fact that the sample hospital was the only healthcare center in the county, this limitation might not have a significant effect on the results. Thirdly, we were only able to extract data from 2015 to 2016, and a longer study period might come up with a more convincing conclusion. Fourthly, potential confounding influences might be neglected, causing selection bias and endogeneity. For instance, patients with different education, occupation, residency, insurance type, and previous years’ household income might end up with different medical costs [27]. Lastly, the study was conducted in a Chinese secondary hospital, the conclusions may not be applicable to other types of healthcare institutions.

## Conclusion

In this study, when compared with FFS, we found GB can curtail health expenditure and improve quality of care. As far as service volume was concerned, LOS and monthly admission declined, while number of drugs per record and record containing antibiotics were not affected. In order to avoid a trade-off effect and advocate a quality-based system, we propose that quality of care be included in evaluations of GB.

## Supplementary information

**Additional file 1.** Supplementary data 1 dependent variables, independent variables and variant

## Data Availability

The datasets used and/or analyzed during the current study are available from the corresponding author on reasonable request.

## Abbreviations

GB: Global Budget
FFS: Fee-For-Service
LOS: Length of Stay
ITS: Interrupted Time Series
WHO: World Health Organization
