# Supplementary material for: The impact of global budget on expenditure, service volume, and quality of care among patients with pneumonia in a secondary hospital in China: a retrospective study
Source: BMC Public Health. 2020 Apr 19;20:522. doi: 10.1186/s12889-020-08619-3 (PMC7168859; doi:10.1186/s12889-020-08619-3)
Supplement: Supplementary file 1 — Additional file 1. Supplementary data 1 dependent variables, independent variables and variant [file 12889_2020_8619_MOESM1_ESM.docx]

Supplementary data 1 dependent variables, independent variables and variant

| **Variable name** | **Description** |
| --- | --- |
| **Dependent variable** |  |
| Expenditure |  |
| Daily health expenditure (RMB) | Continuous, Log transformed |
| Service volume |  |
| Monthly admission | Continuous |
| Length of stay (LOS) | Continuous |
| Number of drugs per record | Continuous |
| Record containing antibiotics | No(0); Yes(1) |
| Quality of care |  |
| Record with multiple antibiotics | No(0); Yes(1) |
| Readmission within 30 days | No(0); Yes(1) |
| **Independent variable** |  |
| Reform | Pre-(0); post-(1) |
| **Covariant** |  |
| Age | [0,5)(1); [5,18)(2); [18,65)(3); [65,95](4) |
| Sex | Male(0); Female(1) |
| Department | Pediatric(0); Internal Medicine(1) |
